# Meta-analysis of factors affecting hyponatremia after spinal cord injury

**DOI:** 10.3389/fneur.2026.1808354

**Published:** 2026-07-03

**Authors:** Jiaojiao Bai, Shihang Cao, Yuzhuo Ma, Xuefei He, Yuanna Zhang, Xi Gao

**Affiliations:** 1 Intensive Care Unit, Honghui Hospital of Xi’an, Jiaotong University, Xi’an, China; 2 Department, Honghui Hospital of Xi’an, Jiaotong University, Xi’an, China

**Keywords:** complete spinal cord injury, hyponatremia, meta-analysis, risk factors, spinal cord injury

## Abstract

**Objective:**

To systematically evaluate the risk factors for hyponatremia in patients with spinal cord injury (SCI) through a meta-analysis, and to provide evidence-based guidance for early identification of high-risk populations and the development of preventive strategies in clinical practice.

**Methods:**

Electronic databases including China National Knowledge Infrastructure (CNKI), Wanfang Database, VIP Database, Chinese Biomedical Literature Database (CBM), PubMed, and Web of Science were searched from their inception to November 10, 2024. Literature screening, data extraction, and quality assessment were independently conducted by two researchers. The Newcastle-Ottawa Scale (NOS) was used to assess the methodological quality of the included studies. Meta-analysis was performed using RevMan 5.3 software.

**Results:**

A total of 14 studies involving 2,729 patients with SCI were included, among whom 1,160 patients developed hyponatremia and 1,569 had normal serum sodium levels. The NOS scores of the included studies ranged from 7 to 8, indicating generally high methodological quality. Meta-analysis results showed that high-level spinal cord injury (OR = 1.71, 95% CI: 1.04–2.81), complete spinal cord injury (OR = 4.96, 95% CI: 3.75–6.57), concomitant traumatic brain injury (OR = 2.70, 95% CI: 1.79–4.07), and the use of assisted ventilation (OR = 3.28, 95% CI: 1.52–7.09) were significant risk factors for hyponatremia in patients with SCI (*p* < 0.05). A funnel plot based on complete spinal cord injury was not completely symmetrical, suggesting a potential risk of publication bias.

**Conclusion:**

Current evidence indicates that high-level spinal cord injury (≤C4), complete spinal cord injury, concomitant craniocerebral injury, and the use of assisted ventilation are significant risk factors for hyponatremia in patients with SCI. Enhanced monitoring and management of these high-risk populations are recommended to facilitate early identification and timely intervention for hyponatremia.

**Systematic review registration:**

The systematic review was registered in PROSPERO (Unique Identifier: CRDCRD42024585004)

## 1 Introduction

Spinal cord injury (1, 2) (SCI) refers to damage to the structure and function of the spinal cord caused by traumatic or non-traumatic factors, which can result in motor, sensory, and autonomic dysfunction below the level of injury. SCI is characterized by a high disability rate and a prolonged rehabilitation period, seriously affecting patients’ quality of life and placing a substantial burden on the healthcare system and society. Epidemiological data indicate that approximately 17,000 new cases of SCI occur annually in the United States, highlighting its significant disease burden (3).

Hyponatremia is one of the most common electrolyte disorders in patients with SCI during hospitalization (4), with a reported incidence ranging from 18.6 to 37.2%, making it the second most common complication after pneumonia (5). The clinical manifestations of hyponatremia following SCI are diverse and may include neuropsychiatric symptoms such as apathy, cognitive impairment, personality changes, and depression. If hyponatremia is not promptly identified and corrected in the early stages, it may lead to serious complication, including cerebral edema, increased intracranial pressure, and even brain herniation. Moreover, inappropriate correction of hyponatremia may result in secondary neurological injuries, such as osmotic demyelination, thereby aggravating neurological dysfunction and potentially becoming life-threatening in severe cases.

In recent years, both domestic and international studies have investigated the mechanisms and influencing factors associated with hyponatremia after SCI, suggesting that injury level and severity, infections, medication use, and endocrine disorder may contribute to its development. However, substantial heterogeneity exists among these studies in terms of study populations, sample sizes, and included variables, leading to inconsistent conclusions. To date, there remains a lack of high-quality, systematic evidence to clearly identify the major risk factors for hyponatremia in patients with SCI. Therefore, the present study aims to systematically evaluate the factors associated with hyponatremia following SCI through a meta-analysis. By synthesizing existing evidence, this study seeks to clarify the risk factors for post-SCI hyponatremia of high-risk populations and the development of targeted prevention and intervention strategies, ultimately improving patient prognosis.

This study has been registered on the PROSPERO platform, registration number CRD42024585004.

## 2 Materials and methods

### 2.1 Literature inclusion and exclusion criteria

Inclusion criteria for the study subjects: ① Source of literature: Relevant studies published domestically and internationally that investigated factors associated with hyponatremia in patients with SCI. ② Type of study: cohort studies or case–control studies, publications were limited to Chinese or English language. ③ Study outcomes: study participants were classified into a hyponatremia group and a normonatremia group based on the occurrence of hyponatremia. Exclusion criteria: ① Duplicate publications. ② Secondary research literature, including systematic reviews, meta-analyses, and narrative reviews. ③ Studies with low methodological quality, incomplete data, or unavailable full-text sources.

### 2.2 Literature search strategy

Electronic searches were conducted in the China National Infrastructure (CNKI), Wanfang Database, VIP Database, Chinese Biomedical Literature Database (CBM), PubMed, and Web of Science. The search period ranged from the inception of each databases to November 10, 2024. Case–control and cohort studies investigating factors associated with the occurrence of hyponatremia occurring after SCI were retrieved. In addition, the reference lists of eligible studies were manually to identify potentially relevant literature. A search strategy combining subject terms and free-text terms was applied. The Chinese search terms included “spinal cord injury”, “hyponatremia”, and “factors” “related factors,” “risk factors,” “influencing factors,” and “association.” The English search terms included “spinal cord injury,” “hyponatremia,” “factor,” “associated factors,” “influence factors,” and “association.”

### 2.3 Data extraction

Two researchers independently screened the literature, assessed the methodological quality of the included studies, extracted relevant data, and performed cross-checking to ensure accuracy. Any disagreements were resolved through discussion, and if consensus could not be reached, a third researcher was consulted for adjudication. During the literature screening process, titles and abstracts were first reviewed, followed by full-text assessment to determine final eligibility. A standardized data extraction from was designed in advance using Microsoft Excel to facilitate data collection. Extracted information included the first author, year of publication, country, study design, total sample size, number of patients with hyponatremia, incidence of hyponatremia, and reported influencing factors.

### 2.4 Quality evaluation

The methodological quality of the included case–control and cohort studies was independently evaluated by two researchers using the Newcastle-Ottawa Scale (NOS) recommended by the Cochrane Collaboration. The NOS assesses study quality (6) across three domains: selection of study populations, comparability between groups, and exposure assessment or outcome evaluation, comprising a total of eight items. The maximum possible score is 9 points, with a total score ≥7 indicating high methodological quality. In cases of disagreement during quality assessments, the final judgment was reached through discussion between the two researcher or, if necessary, by consulting with a third researcher.

### 2.5 Statistical analysis

All statistical analyses were performed using RevMan 5.3 software. In the quantitative synthesis, continuous variables are expressed as mean differences (MDs) with 95% confidence intervals (CIs), while dichotomous variables were expressed as odds ratios (ORs) with 95% confidence intervals (CIs). Statistical heterogeneity among studies was assessed using the I^2^statistic and the chi-square test. When *p* > 0.10 and I^2^ < 50%, heterogeneity was considered low, and a fixed-effects model was applied. When *p* ≤ 0.10 and I^2^ ≥ 50%, significant heterogeneity was assumed, and a random-effects model was used. Sensitivity analysis were conducted by switching between fixed-effects and random-effects models or by sequentially excluding studies with greater weight to assess the stability of the pooled results. If certain influencing factors could not be quantitatively pooled, a descriptive analysis was performed. In addition, funnel plots were constructed to assess potential publication bias for influencing factors included in 10 or more studies.

## 3 Results

### 3.1 Literature search results

**Figure 1:**
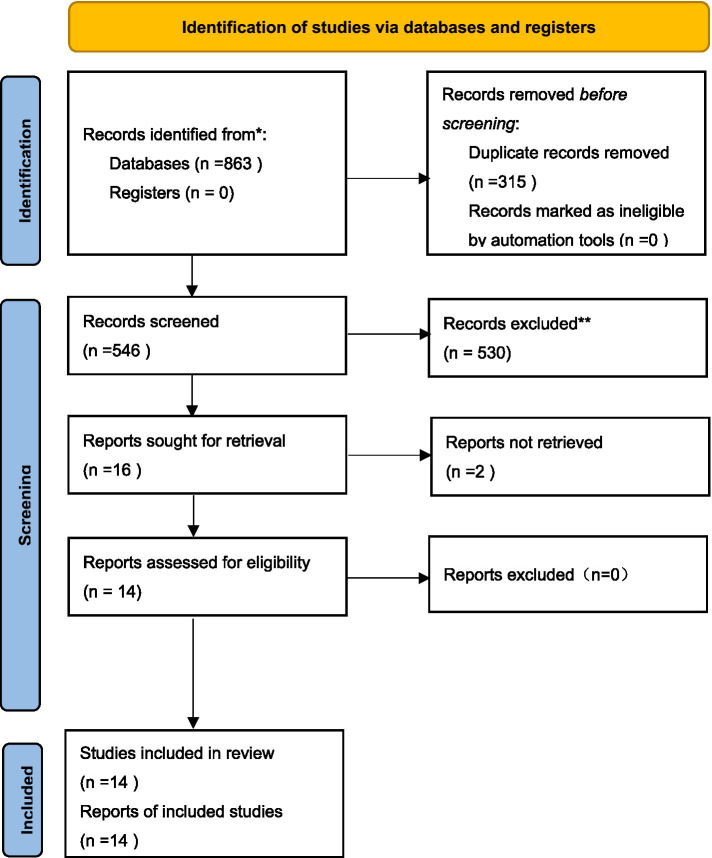
Flow chart of study selection.

A total of 863 potentially relevant studies were identified through the literature search, including 42 from PubMed, 59 from Web of Science, 224 from China National Knowledge Infrastructure (CNKI), 180 from the Chinese Biomedical Literature Database (CNM), 230 from Wanfang Database, and 128 from VIP Database. After multiple rounds of screening, 14 studies were ultimately included in the meta-analysis, of which 10 were published in Chinese (1, 7–15) and 4 in English (16–19). The literature screening process and selection results are presented in Figure 1.

### 3.2 Basic characteristics and quality assessment of included literature

A total of 14 studies were included in the present analysis, comprising 12 case–control studies and 2 cohort studies. The total sample size was 2,729 patients, of whom 1,160 developed hyponatremia after spinal cord injury and 1,569 did not develop hyponatremia. According to the Newcastle-Ottawa Scale (NOS), the quality scores of the included studies ranged from 7 to 8, indicating overall good methodological quality. The basic characteristics of the included studies are summarized in Table 1, and the results of the quality assessment are presented in Table 2.

**Table 1:** Basic characteristics of the included literature (*n* = 12).

Study	Country	Total number of cases (cases)	Number of cases of hyponatremia	Incidence of hyponatremia (%)	Exposure factors	Study design	Hyponatremia criteria
Jin, 2023 (1)	China	230	128	55.7	1.2.3.4.6.8.10.16	Case–control	Serum Na < 135 mmol/L
Deng, 2018 (8)	China	67	49	73.13	1.2.3.4.5.6.8.9.12	Case–control	Serum Na < 135 mmol/L
Li, 2017 (13)	China	49	26	53	1.2.3.4.6.16	Case–control	Serum Na < 135 mmol/L
Yan, 2016 (9)	China	369	148	40.1	1.2.3.6.9.10.12.14	Case–control	Serum Na < 135 mmol/L
Bao, 2013 (6)	China	121	53	43.8	1.2.3.4.5.6.7.8.9.11.17.19	Case–control	Serum Na < 135 mmol/L
Tang, 2012 (12)	China	62	31	50	2.3.4.10.13.16	Case–control	Serum Na < 135 mmol/L
Li, 2011 (7)	China	68	17	25	1.2.3.4.8.9.10	Cohort study	Serum Na < 135 mmol/L
Kang, 2008 (17)	China	69	37	53.62	1.2.3.5.8.9.12	Cohort study	Serum Na < 135 mmol/L
Tan, 2005 (10)	China	112	89	79.4	1.2.3.4.5.8.10	Case–control	Serum Na < 135 mmol/L
Cholavech, 2022 (15)	Thailand	123	54	44	1.2.3.4	Case–control	Serum Na < 135 mmol/L
Hiroyuki, 2019 (16)	Japan	213	85	40	1.2.3.4.8.15.16.17.18.19.20	Case–control	Serum Na < 135 mmol/L
PW Song, 2017 (14)	China	270	82	30.4	1.2.4.10.15.16.19.20	Case–control	Serum Na < 135 mmol/L

Risk factor: 1 = Age; 2 = Gender; 3 = Spinal cord injury level; 4 = ASIA grading; 5 = Time from injury to hospital stay (course of illness); 6 = Whether to use glucocorticoids; 7 = Whether to use mannitol; 8 = Whether there is a head injury; 9 = Whether there is a high fever; 10 = Whether it assists breathing; 11 = Whether it is paralyzed; 12 = Cause of injury; 13 = Body mass index (BMI); 14 = Treatment method (conservative/Surgery); 15 = hematocrit (HCT); 16 = hemoglobin (HGB); 17 = leukocytes (WBC); 18 = plasma albumin; 19 = potassium concentration; 20 = chloride concentration.

**Table 2:** Quality evaluation results of case–control studies and cohort studies (*n* = 12).

Studies were included	Selection of study subjects				Comparability between groups		Measurement of exposure factors			NOS score
	(1)	(2)	(3)	(4)	(5A)	(5B)	(6)	(7)	(8)	
Jin, 2023 (1)	1	1	0	1	1	0	1	1	1	7
Deng, 2018 (8)	1	1	0	1	1	0	1	1	1	7
Li, 2017 (13)	1	1	0	1	1	1	1	1	1	8
Yan, 2016 (9)	1	1	0	1	1	0	1	1	1	7
Bao, 2013 (6)	1	1	0	1	1	0	1	1	1	7
Tang, 2012 (12)	1	1	0	1	1	0	1	1	1	7
Li, 2011 (7)	1	1	0	1	1	1	1	1	1	8
Kang, 2008 (17)	1	1	0	1	1	1	1	1	1	8
Tan, 2005 (10)	1	1	0	1	1	0	1	1	1	7
Cholavech, 2022 (15)	1	1	0	1	1	1	1	1	1	8
Hiroyuki, 2019 (16)	1	1	0	1	1	1	1	1	1	8
PW Song, 2017 (14)	1	1	0	1	1	1	1	1	1	8

(1) 1. Appropriateness of case determination, 2. Representativeness of cases, 3. Selection of controls, 4. Identification of controls, 5A. Study controlled for the most important confounders, 5B. Study controlled for any other confounders, 6. Identification of exposure factors, 7. Identification of case and control exposures using the same methodology, 8. Non-response rate.

(2) 5A = Study controlled for the most important confounders; 5B = Study controlled for any other confounders.

### 3.3 Meta-analysis

#### 3.3.1 Meta-analysis results

Among the 14 included studies, high-level spinal cord injury (≤C4), complete spinal cord injury [ASIA grade A, i.e., complete injury (20)], concomitant traumatic brain injury, and the use of assisted ventilation were identified as risk factors for hyponatremia following spinal cord injury. The pooled results are summarized.

##### 3.3.1.1 High-level spinal cord injury (≤C4)

**Figure 2:**
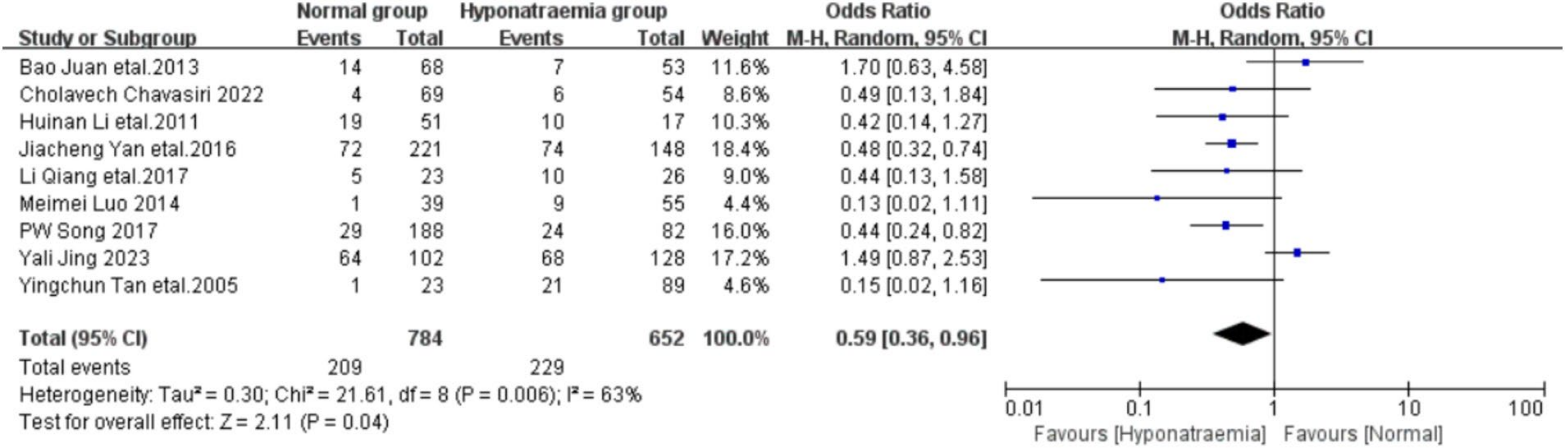
Forest plot of high-level spinal cord injury (≤C4).

Nine studies reported the association between high-level spinal cord injury and the occurrence of hyponatremia after SCI. Significant heterogeneity was observed among the studies (I2 = 63%, *p* < 0.10). Therefore, a random-effects model was applied. The pooled result showed that high-level spinal cord injury was a significant risk factor for hyponatremia following SCI (OR = 1.71, 95% CI: 1.04–2.81, *p* = 0.04), as shown in Figure 2.

##### 3.3.1.2 Complete spinal cord injury

**Figure 3:**
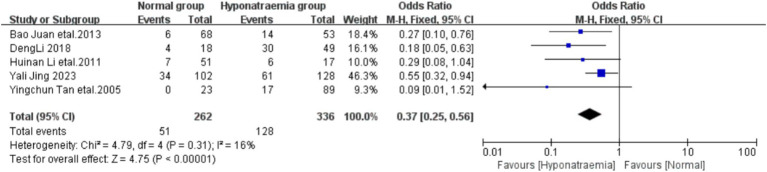
Forest plot of complete spinal cord injury.

Thirteen studies examined the relationship between complete spinal cord injury and hyponatremia after SCI. Low heterogeneity was detected among the studies (I^2^ = 31%, *p* > 0.10); thus, a fixed-effects model was used, the meta-analysis demonstrated that complete spinal cord injury was a significant risk factor for hyponatremia after SCI (OR = 4.96–95% CI: 3.75, 6.57, *p* < 0.01), as shown in Figure 3.

##### 3.3.1.3 Combined craniocerebral injury

**Figure 4:**
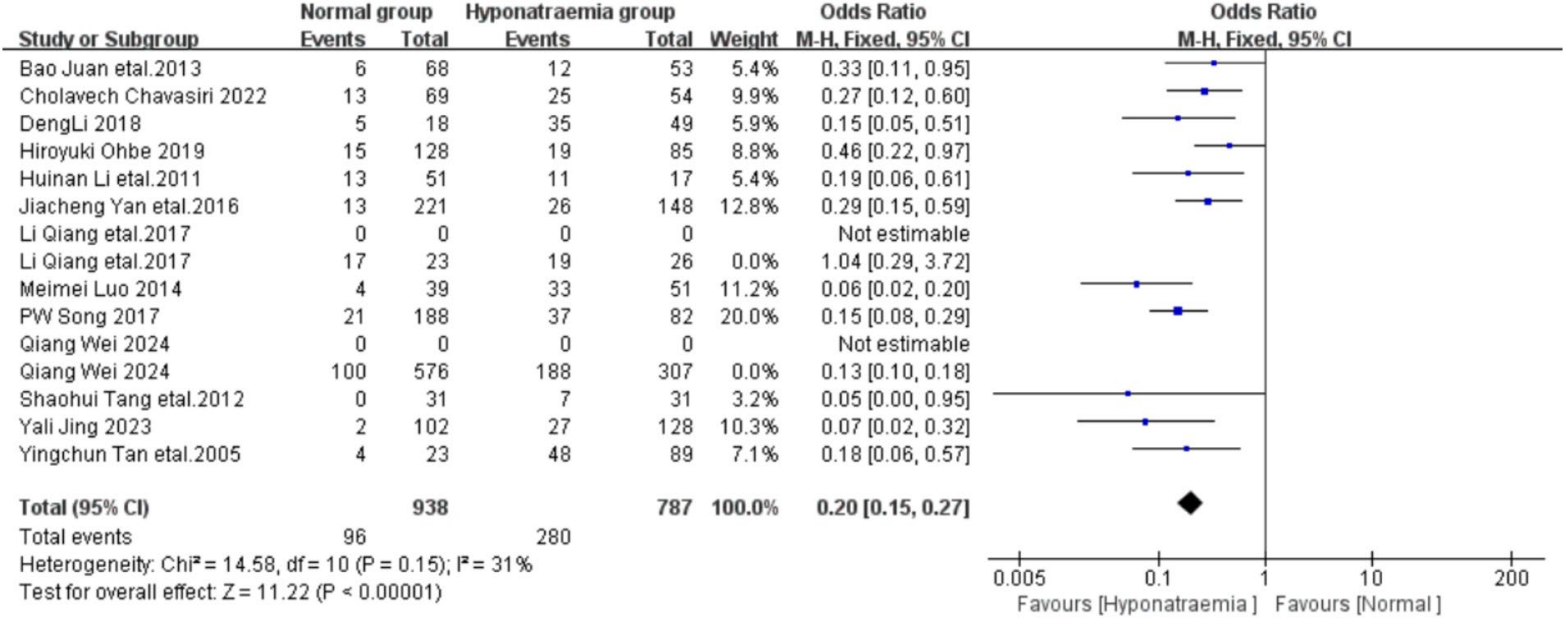
Forest plot of concomitant craniocerebral injury.

Five studies reported the association between concomitant traumatic brain injury and the occurrence of hyponatremia after SCI. The included studies showed low heterogeneity (I2 = 16%, *p* > 0.10). A fixed-effects model was applied, and the pooled analysis indicated that concomitant traumatic brain injury was a significant risk factor for hyponatremia following SCI (OR = 2.70, 95% CI: 1.79–4.07, *p* < 0.01), as shown in Figure 4.

##### 3.3.1.4 Assisted breathing

**Figure 5:**
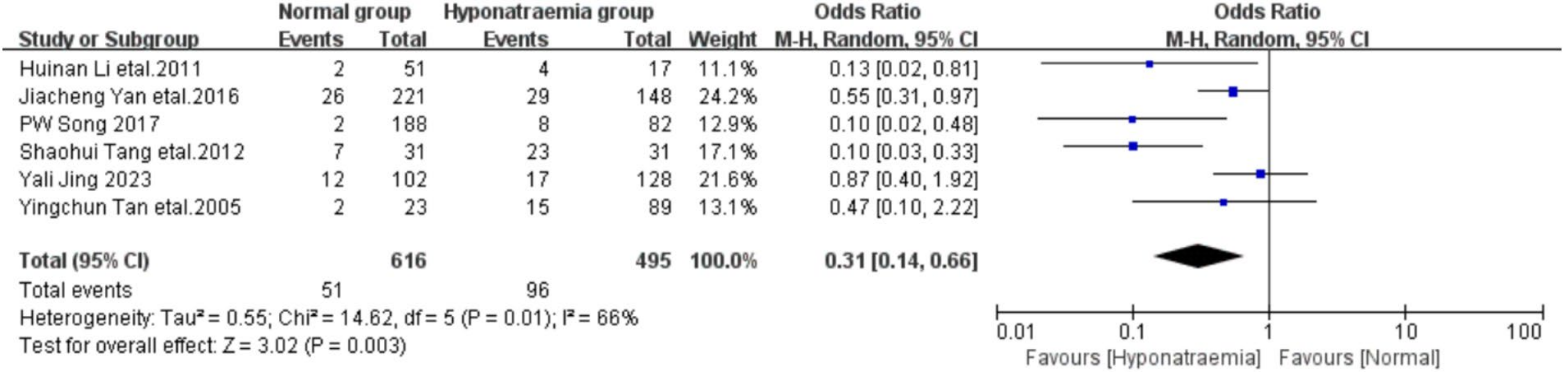
Forest plot of assisted ventilation.

Six studies investigated the relationship between assisted ventilation and the occurrence of hyponatremia after SCI. Significant heterogeneity was observed (I^2^ = 66%, *p* < 0.10); therefore, a random-effects model was used, the pooled results showed that assisted ventilation was a significant risk factor for hyponatremia following SCI (OR = 3.28, 95% CI:1.52–7.09, *p* < 0.01), as shown in Figure 5.

#### 3.3.2 Heterogeneity test

Substantial heterogeneity was observed among studies for age, male sex, high-level spinal cord injury (≤C4), corticosteroid use, high fever, and assisted ventilation (I^2^ ≥ 50%); therefore, a random-effects model was applied for these factors. In contrast, complete spinal cord injury, concomitant traumatic brain injury, and paralysis showed low heterogeneity among studies (I^2^ < 50%), and a fixed-effects model was used for the pooled analysis. The detailed results are presented in Table 3.

**Table 3:** Meta-analysis of factors affecting hyponatremia after spinal cord injury (*n* = 12).

Exposure factors	Included literature (article)	Heterogeneity test		Effect model	Results of meta-analysis	
		I^2^ (%)	*P*		OR/MD (95%CI)	*P*
Age	6 (1, 7, 9–11, 14)	87	<0.001	Random	1.35 (−3.47, 6.17)	0.58
Male	10 (6, 8–10, 12–16)	55	0.02	Random	1.21 (0.81, 1.82)	0.09
High spinal cord injury (≥C_4_)	8 (1, 6, 7, 9, 10, 13–15)	64	0.007	Random	1.59 (0.97, 2.62)	0.07
Complete spinal cord injury	11 (1, 6–10, 12–16)	35	0.12	Fixed	4.21 (3.18, 5.57)	<0.001
Glucocorticoids	5 (6, 8, 9, 13)	63	0.03	Random	0.84 (0.48, 1.46)	0.53
Concomitant head injury	5 (1, 6–8, 10)	16	0.31	Fixed	2.70 (1.79, 4.07)	<0.001
Hyperpyrexia	3 (6, 8, 9)	90	<0.001	Random	0.76 (0.12, 5.02)	0.78
Assisted breathing	6 (1, 7, 9, 10, 12, 14)	66	0.01	Random	3.28 (1.52, 7.09)	0.003
Paralysis	2 (6, 17)	28	0.24	Fixed	2.25 (0.89, 5.70)	0.09

#### 3.3.3 Sensitivity analysis

##### 3.3.3.1 Replacement effect model

Sensitivity analysis were performed by applying both fixed-effects and random-effects models to calculate the pooled odds ratios (ORs) and 95% confidence intervals (CIs) for each influencing factor. The results showed that, expect for sex (male), high-level spinal cord injury (≤C4), and high fever, the pooled ORs and corresponding 95% CIs for the remaining factors were largely consistent between the two models, indicating that the overall results of the meta-analysis were stable and reliable in Table 4.

**Table 4:** Sensitivity analysis.

Exposure factors	Before sensitivity analysis			After sensitivity analysis		
	Effect size (MD/OR)	95%CI	*P*	Effect size (MD/OR)	95%CI	*P*
Age	1.35	−3.47, 6.17	0.58	−0.06	−0.90, 0.77	0.88
Male	1.21	0.81, 1.82	0.09	1.12	0.88, 1.43	0.35
High spinal cord injury (≥C_4_)	1.59	0.97, 2.62	0.07	1.53	1.19, 1.97	0.001
Complete spinal cord injury	4.21	3.18, 5.57	<0.001	4.06	2.79, 5.91	<0.001
Glucocorticoids	0.84	0.48, 1.46	0.53	0.87	0.64, 1.17	0.36
Concomitant head injury	2.70	1.79, 4.07	<0.001	2.83	1.71, 4.70	<0.001
Hyperpyrexia	0.76	0.12, 5.02	0.78	1.19	0.82, 1.74	0.37
Assisted breathing	3.28	1.52, 7.09	0.003	2.43	1.66, 3.55	<0.001
Paralysis	2.25	0.89, 5.70	0.09	2.31	0.73, 7.27	0.15

##### 3.3.3.2 Sensitivity analysis by sequential exclusion

For influencing factors with I^2^ ≥ 50% and more than two included studies, sensitivity analysis was further conducted using a sequential exclusion approach. The results demonstrated that, after excluding certain studies, the heterogeneity of two factors—high-level spinal cord injury and complete spinal cord injury—was markedly reduced. Specifically, the study by Jing Yali et al. (1) was identified as the primary source of heterogeneity for the high-level spinal cord injury, and exclusion of this study resulted in a substantial decrease in heterogeneity (I^2^ = 23%, *p* = 0.25). Similarly, the studies by Li Qiang, Qiang Wei, and Hiroyuki Ohbe (8, 18, 19) were identified as the main sources of heterogeneity for complete spinal cord injury, and their exclusion led to a marked reduction in heterogeneity (I^2^ = 7%, *p* = 0.38). For the remaining influencing factors, no significant change in heterogeneity was observed following sequential exclusion, indicating good robustness of the pooled results.

#### 3.3.4 Publication bias test

**Figure 6:**
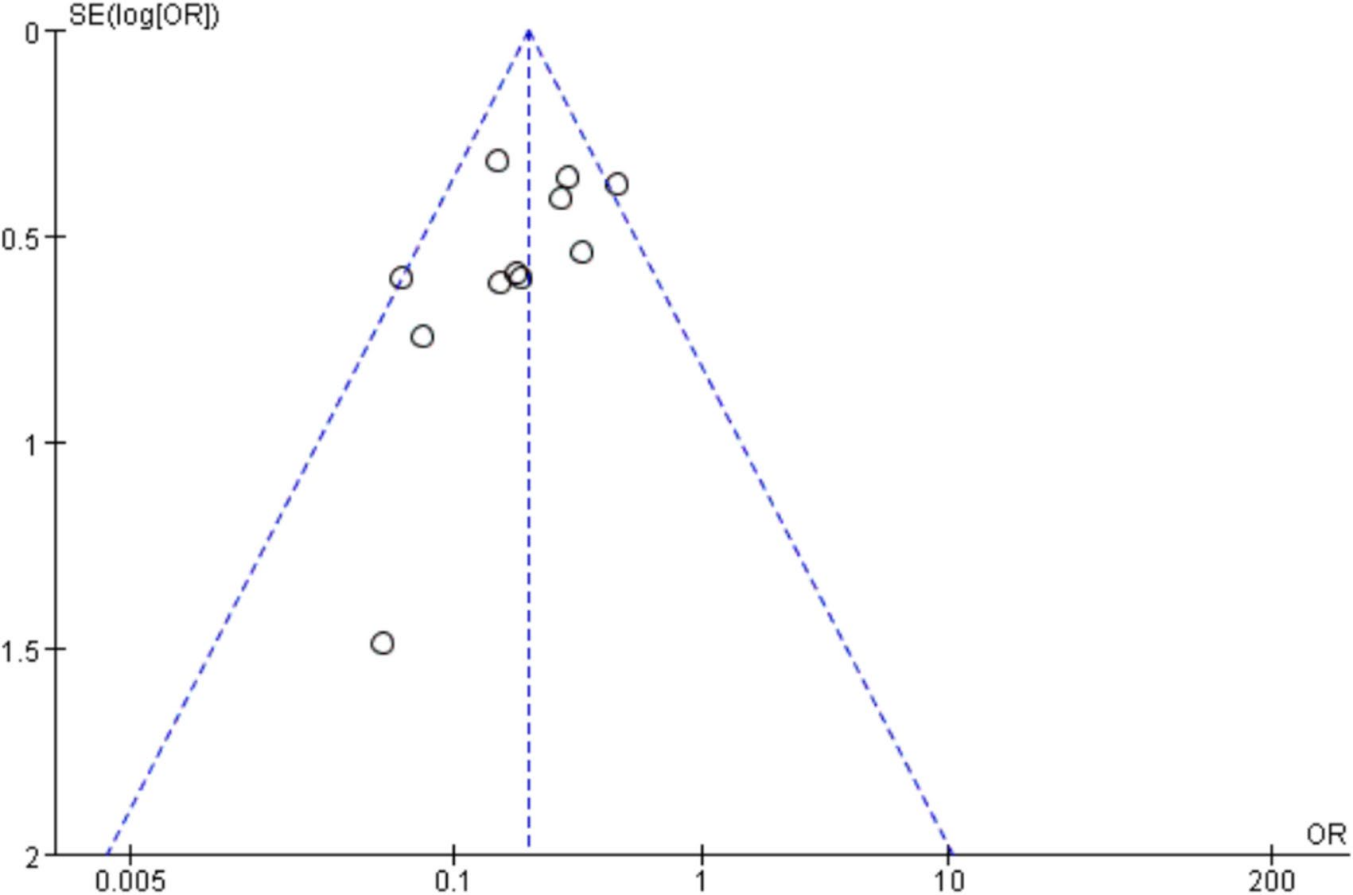
Funnel chart of completeness damage factors.

**Figure 7:**
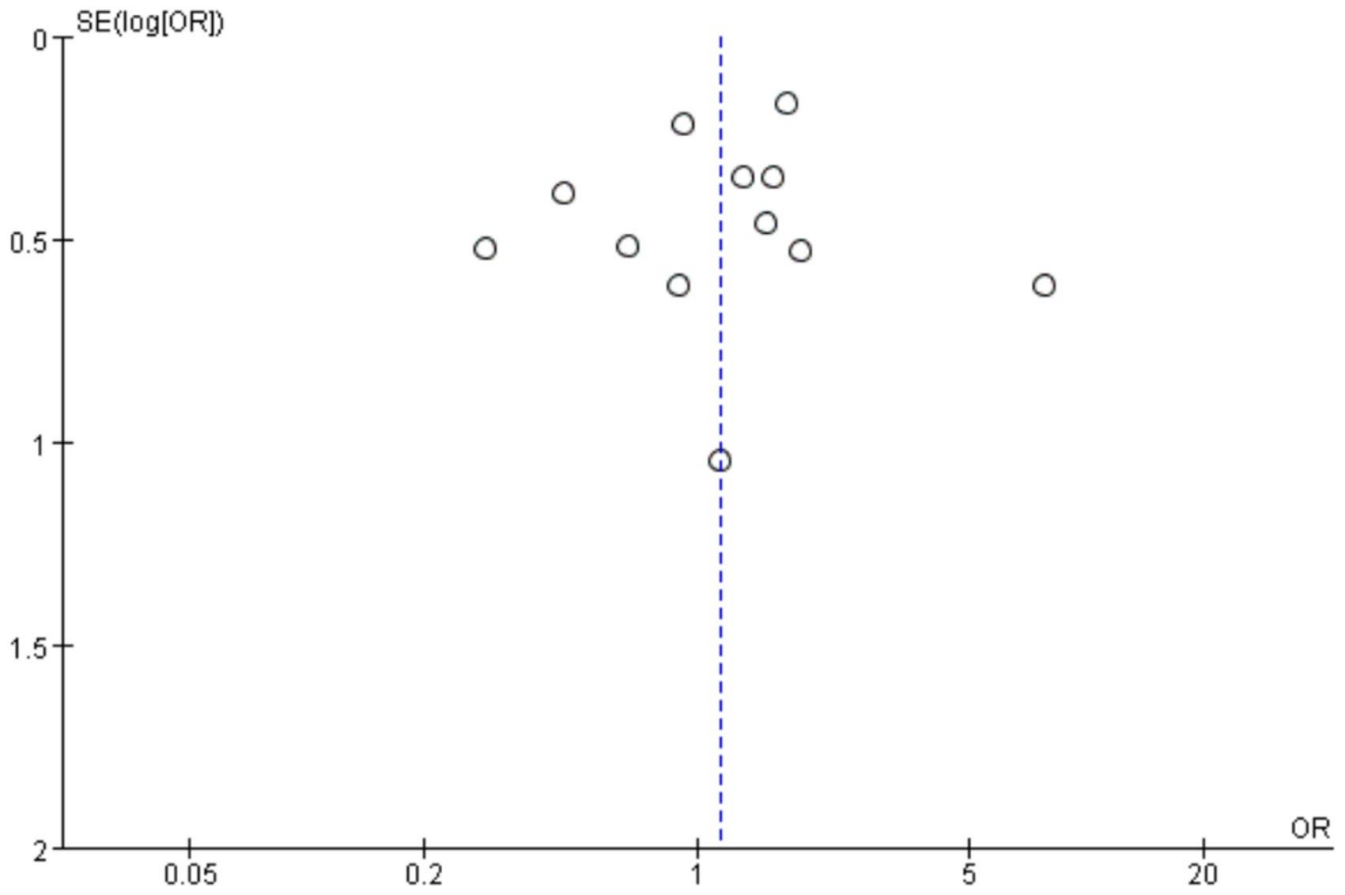
Funnel plot for gender factors.

For risk factors included in 10 or more studies, funnel plots were constructed to assess potential publication bias. When complete spinal cord injury was used as the study factor, the funnel plot showed a generally symmetrical distribution of points, indicating no obvious evidence of publication bias among the included studies (Figure 6). In contrast, when sex (male) was used as the study factor, the funnel plot exhibited an asymmetric distribution of points on both sides, suggesting the possible presence of publication bias (Figure 7).

#### 3.3.5 Descriptive analysis

A descriptive analysis was performed for influencing factors that could not be quantitatively pooled in the meta-analysis across the 14 included studies. Tang Shaohui et al. (9), reported that concomitant pulmonary infection (OR = 16.185, 95% CI: 2.472–93.280), upper cervical spinal cord injury (C1-2) (OR = 26.254, 95% CI: 1.957–231.513), and hypoproteinemia (OR = 11.889, 95% CI: 2.212–98.938) were significantly associated with hyponatremia following spinal cord injury (*p* < 0.05). Jing Yali et al. (1) founded that increased 24-h urine volume was a risk factor for hyponatremia after spinal cord injury (OR = 1.004, 95% CI: 1.003–1.005), whereas spinal cord injury decompression surgery was identified as a protective factor against the development of hyponatremia (OR = 0.461, 95% CI: 0.250–0.851) (*p* < 0.05). In addition, PW Song et al. (17) reported that hypotension was a significant contributing factor to hyponatremia following spinal cord injury (OR = 5.632, 95% CI: 1.932–16.414) (*p* < 0.05).

## 4 Discussion

The prevalence of hyponatremia in patients with acute SCI is substantially higher than that in the general medical or surgical patient population. If hyponatremia is not promptly recognized and appropriately corrected, it may lead to serious complications and can even be life-threatening. The development of hyponatremia following SCI is influenced by multiple factors. The findings of this meta-analysis indicate that high-level spinal cord injury, complete spinal cord injury, concomitant traumatic brain injury, and the use of assisted ventilation are independent risk factors for the occurrence of hyponatremia after SCI.

### 4.1 Analysis of factors affecting hyponatremia after SCI

#### 4.1.1 High cervical spinal cord injury (≤C4)

The results of this meta-analysis indicate that high-level spinal cord injury (≤C4) is a significant risk factor for the development of hyponatremia in patients with SCI. This finding is generally consistent with previous studies. The neurological level of injury is considered an important predictor of prognosis in patients with acute cervical spinal cord injury (21). Yan Jiacheng et al. (12) reported that the incidence of hyponatremia was significantly higher in patients with high-level spinal cord injuries than in those with lower-level injuries, suggesting a close association between injury level and the risk of hyponatremia.

However, some studies have suggested that high-level spinal cord injury is not an independent risk factor for hyponatremia (1). Such discrepancies may be attributed to differences in injury characteristics, sample sizes, and heterogeneity in study design among the included studies. Despite these inconsistencies, given the severity of illness and the high incidence of complications in patients with high-level spinal cord injuries, enhanced and dynamic monitoring of serum sodium levels is recommended in clinical practice to facilitate early detection and timely correction of hyponatremia, thereby improving patient outcomes.

#### 4.1.2 Complete spinal cord injury (ASIA grade A)

This present study found that complete spinal cord injury is an important risk factor for hyponatremia in patients with SCI, with a significantly higher risk compared with incomplete spinal cord injury (OR = 4.21). This finding suggests that the occurrence of hyponatremia is closely associated with the severity of spinal cord injury, which is consistent with the results reported by Y Nakao et al. (22). Li Cheng et al. (23) further demonstrated that the incidence of hyponatremia was significantly higher in patients with ASIA grade A injury than in those with ASIA grades B, C, and D. In addition, Feng Wei et al. (24) observed in animal experiments that the number of spinal cord axons in rats was significantly reduced under hyponatremic conditions, providing pathological evidence to support the association between hyponatremia and the severity of spinal cord injury.

Therefore, for patients with ASIA grade A spinal cord injury, clinicians should remain highly vigilant regarding the risk of hyponatremia. Early and close monitoring of serum sodium levels after injury, along with timely preventive interventions, is recommended to reduce the occurrence of hyponatremia and improve clinical outcomes.

#### 4.1.3 Combined craniocerebral injury

Craniocerebral injury can directly disrupt central regulatory mechanisms of water and sodium metabolism, thereby predisposing patients to electrolyte imbalance. The results of the present study indicate that patients with spinal cord injury (SCI) combined with traumatic brain injury have a 2.70-fold increased risk of developing hyponatremia compared with those without craniocerebral injury. This finding is consistent with the results reported by Deng Li et al. (25) and other reflects a relatively unified consensus in both domestic and international studies (14, 26, 27).

The underlying mechanism may be explained as follows. First, traumatic brain injury may stimulate or damage the hypothalamic–pituitary axis and other neuroendocrine regulatory centers (28), resulting in cerebral salt-wasting syndrome or syndrome (CSWS) or syndrome of inappropriate antidiuretic hormone secretion (SIADH), which in turn leads to central hyponatremia. In addition, Zhang Li et al. (29) reported that cervical spinal cord injury can inhibit sympathetic nervous system activity, causing hypotension and reduced renal perfusion. These changes may trigger renal natriuretic and diuretic responses, further increasing sodium loss and aggravating the risk of hyponatremia. Therefore, in patients with SCI complicated by craniocerebral injury, enhanced monitoring and management of water and sodium metabolism are essential to prevent electrolyte disturbances and further clinical deterioration.

#### 4.1.4 Assisted breathing

The results of this meta-analysis demonstrated that the use of assisted breathing in patients with spinal cord injury (SCI) was associated with a significantly increased risk of hyponatremia (OR = 3.28). Similar findings have been reported by Wang Xue (30) and Li Weiling (31), who also suggested mechanical ventilators is closely related to the occurrence of hyponatremia. The requirement for assisted respiratory support usually reflects greater injury severity. Such patients often require prolonged bed rest and intensive care, which increases their susceptibility to complications such as pulmonary and urinary tract infections. Previous studies have shown that various infectious conditions are closely associated with the development of hyponatremia (32). In addition, mechanical ventilation itself may promote hyponatremia by stimulating the secretion of antidiuretic hormone and altering neuroendocrine regulation. Therefore, for SCI patients requiring assisted respiratory support, standardized and dynamic electrolyte monitoring protocols should be established to enable early detection and timely correction of hyponatremia, thereby improving clinical outcomes.

### 4.2 Limitations of this study, prospects, and suggestions for future research

This study has several limitations. First, only literature published in Chinese and English was included, which may have led to incomplete retrieval of relevant studies and potential language bias. Second, the number of included was relatively limited, and some studies had small sample sizes, which may have reduced the representativeness of the findings and the overall strength of the evidence. Finally, although this study identified factors such as mannitol use and causes of injury as potential contributors to hyponatremia following SCI, the limited number of eligible studies prevented these variables from being included in the meta-analysis. Future research should focus on large-sample, multicenter, prospective studies to further validate the refine these findings. In addition, several directions merit further investigation. First, the scope of literature searches should be expanded the comprehensiveness and robustness of the evidence base. Second, the causal relationships between mannitol use, fluid management strategies, different injury mechanisms, and the development of hyponatremia should be explored in greater depth. Third, by integrating clinical characteristics with laboratory, future studies may aim to construct and validate risk prediction models for hyponatremia following SCI, thereby, providing evidence-based guidance for early identification and individualized clinical interventions.

## 5 Conclusion

In summary, current evidence suggests that high-level spinal cord injury (≤C4), complete spinal cord injury, concomitant traumatic brain injury, and the use of assisted ventilation are significant risk factors for the development of hyponatremia in patients with SCI. These findings provide evidence-based support for clinical populations to facilitate early identification of high-risk populations and the implementation of targeted preventive and therapeutic interventions. However, given the limitations in the number and methodological quality of the included studies, these conclusions should be interpreted with caution and require further validation through high-quality, large-sample research.

## Data Availability

The original contributions presented in the study are included in the article/supplementary material, further inquiries can be directed to the corresponding authors.
